# The Translation and Validation of Patient-Centered Outcomes Questionnaire Into Arabic: A Chronic Periodontitis Patient-Based Study

**DOI:** 10.7759/cureus.52786

**Published:** 2024-01-23

**Authors:** Khalid Alkhurayji, Sultan Aldakhil, Nouf Alhrabi, Mohammed Aljuaid, Faisal Asiri

**Affiliations:** 1 Health Information Management and Technology, Imam Abdulrahman Bin Faisal University, Riyadh, SAU; 2 Oral and Dental Health, Dental Center, Prince Sultan Military Medical City, Riyadh, SAU; 3 Restorative & Prosthetic Dental Sciences, College of Dentistry, King Saud Bin Abdulaziz University for Health Sciences, Riyadh, SAU; 4 Health Administration, College of Business Administration, King Saud University, Riyadh, SAU; 5 Dental Health, College of Medical Applied Sciences, King Saud University, Riyadh, SAU

**Keywords:** reliability, validation, translation, patient-centered, periodontitis

## Abstract

Background

The patient-centered outcomes questionnaire (PCOQ) is a self-report questionnaire that aims to assess four fundamental domains (pain, fatigue, distress, and interference) on an 11-point numerical rating scale from 0 to 10 in chronic diseases. The implementation of this tool will help assess chronic diseases; hence, this study aimed to translate the PCOQ to a sample of periodontitis patients.

Methodology

This study went through the content validity index. Arabic PCOQ used Cronbach’s alpha for reliability with 300 participants. From July to August 2023, patients with periodontitis visiting an outpatient dental center in Riyadh were invited to participate in the study. The language, content, and structure of the questionnaire were appropriate, and with forward and backward translation, external entity translation was implemented.

Results

Regarding the participants’ gender, the number of males was higher at 61%. Regarding age distribution, 50.7% of the participants were between 30 and 50 years old. A clarity score of 95.2% and a representativeness score of 97.3% were reported in the content validity analysis. The Cronbach’s alpha of the Arabic PCOQ questionnaire was 0.85, and the subscales ranged between 0.68 and 0.93.

Conclusions

The translated version of the Arabic PCOQ is a valid tool to be used in Arab countries. Nonetheless, this instrument can provide insights for healthcare professionals, policymakers, and managers to improve patient satisfaction and healthcare system delivery.

## Introduction

The definition and classification of periodontitis are still not definitive; however, one of the most accepted definitions is that it is a chronic inflammatory condition of the gums and its surroundings accompanied by a deterioration of the connective tissues and alveolar bone that, in some cases, can lead to tooth loss [1]. Unfortunately, in most cases, the disease remains untreated because there is little evidence of discomfort in the early stages, with recognizable symptoms appearing only after extensive disease progression. Moreover, it has been observed that there is a strong link between periodontal disease and systemic disorders, with untreated and neglected periodontal disease increasing the risk of premature birth, cardiovascular disease, and other diseases [2,3]. Hence, not only early detection and treatment are required but also the development of an all-encompassing strategy for periodontal disease prevention is needed [4]. This condition is recognized globally as a burden on the healthcare system. Periodontal disease causes tooth loss if not treated promptly, and untreated periodontitis may hinder chewing ability, word pronunciation, and aesthetic function [5,6]. These findings reduce the health-related quality of life of a wide range of populations, including the elderly, adults, pregnant women, and workers [7,8]. Common impacts on quality of life include decreased food consumption due to impaired chewing function, the development of gastrointestinal problems and nutritional imbalances, and the inability to talk and participate socially. Pain due to periodontal disease can also cause work absenteeism and sleep difficulties, leading to financial loss. Thus, assessing health-related quality of life for the prevention, treatment, and management of periodontal disease is required to design interventions and monitor disease burden. However, there is insufficient quantitative research worldwide into the lives of periodontal patients [9]. In Saudi Arabia, 30.1% to 50.8% of the adult population aged between 25 and 55 years are reported to be affected by chronic periodontal disease [10]. Recent studies [11,12] have investigated a broad range of satisfaction indicators, generally known as patient-centered outcomes. A few limitations of the patient-centered outcome literature are worth mentioning. Little is known about the specific elements that chronic pain patients believe are necessary for successful therapy, or whether these factors change among pain patient demographics. The patient-centered outcomes questionnaire (PCOQ) is a self-report questionnaire that rates four dimensions relevant to chronic pain populations (pain, fatigue, distress, and interference) on an 11-point numerical rating scale (NRS) of 0 to 10. The PCOQ asks patients to rate their usual levels in each of these four areas, as well as what levels they perceive to be a minimally successful treatment outcome, what levels they want, and what levels they expect after treatment. Patients rate the importance of treating each of these four domains. To our knowledge, no study has investigated the impact of periodontal disease in Saudi Arabia using the PCOQ. Hence, this study aimed to report the translation and validation of the PCOQ on periodontitis patients in Saudi Arabia. Due to the anticipated increase in the number, patients from Saudi Arabia are suitable candidates for such studies [13].

## Materials and methods

Instrument

**Figure 1:**
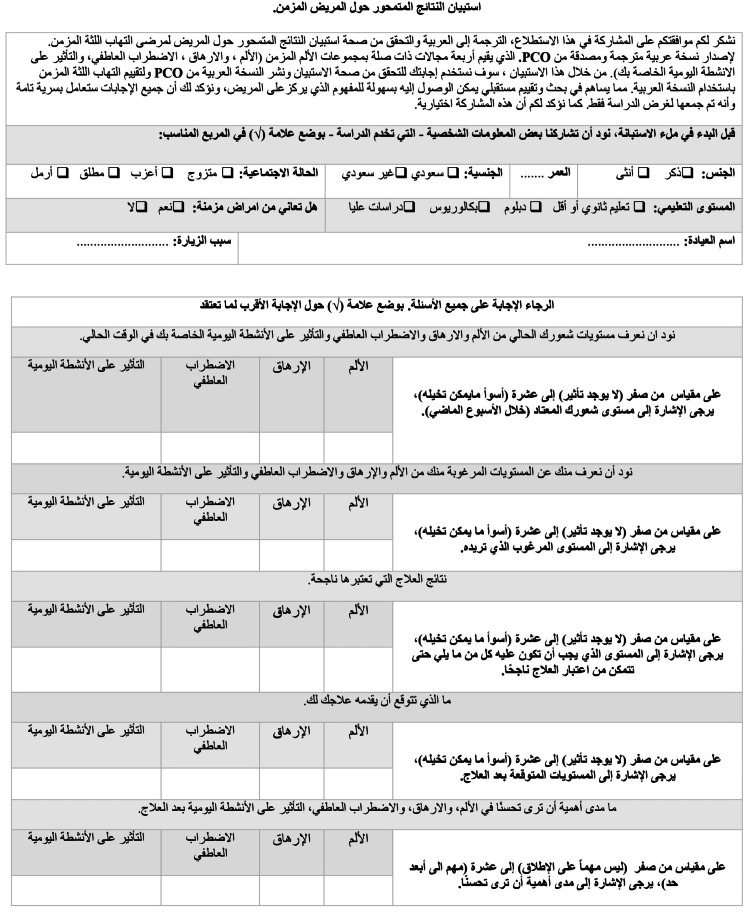
The final version of the patient-centered outcome questionnaire in Arabic.

The PCOQ is a patient-centered questionnaire that rates the dimensions important to chronic periodontitis patients in terms of interference, pain, fatigue, and distress on an 11-point NRS ranging from 0 to 10 [14]. The PCOQ provides patients with scores of their experience regarding their usual levels in these dimensions, the level they perceive to consider successful treatment, in addition to what level they desire, and the levels they expect to achieve post-treatment. Chronic patients rate the importance of treating each of the four domains. The first version was developed after a group of Arabic and English experts translated the list of items into Arabic. The second version was developed after an external panel of researchers participated in assessing the translated version. After confirmation of the second translation into Arabic, an independent professional translator fluent in both Arabic and English translated the instrument into English. As a result, a final third version was provided after only minor alterations were reported. Of note, the translator was not aware of the original English version of the tool. Figure 1 shows the final version of the Arabic PCOQ.

Forward and Backward translation

The recommended World Health Organization multistep approach for translating instruments published in English into foreign languages was used [15]. A group of native Arabic speakers translated the list of items into Arabic. Then, evaluation and comparison to English were performed, as further discrepancies were discussed and resolved. This process took more than a week and resulted in the initial draft of the Arabic PCOQ. Subsequently, an external panel of researchers participated in assessing the translated version. Special consideration was given to the medical terms, as they may be interpreted differently due to dialectical variances in Arabic-speaking countries. Then, an independent professional translator fluent in both Arabic and English translated the instrument into English, and only minor alterations were reported.

Content validity

The content validity index was used to assess the language, content, and structure of the Arabic PCOQ which were adequate for assessing patient-centered outcomes for periodontitis patients. Clarity and representativeness were the two main domains on which the evaluation was done. The content validity index and the scale content validity index were evaluated separately. The scale content validity index is the mean of all reviewer values, while the item content validity index is the proportion of reviewers who evaluated an item as very or highly relevant. An agreement rate of 78% or higher shows good content validity [16].

Validation with chronic periodontitis patients

From July to August 2023, patients with chronic periodontitis visiting an outpatient dental center in Riyadh were invited to participate in the study. Patients were reached in the waiting area of the periodontal clinics and requested to fill out the questionnaire in person after checking the health information management system to confirm that the patient had chronic periodontitis. Our convenience sample strategy yielded 300 individuals. The sample size was calculated through statistical software to be a minimum of 142 participants by considering an alpha error of probability of 0.05, an effect size of 0.2, and a study power of 0.8.

Inclusion and exclusion criteria

Chronic periodontitis patients visiting the periodontal clinic were included in this study. Patients visiting the periodontal clinic who were not diagnosed with chronic periodontitis were excluded.

Statistical analysis

SPSS version 25 (IBM Corp., Armonk, NY, USA) was used for data analysis. The processes comprised a description of PCOQ domains, items, and all subscales. Additionally, participant demographics were collected. PCOQ items were measured as standard deviations and mean, following the tool’s instructions [14]. The demographic features of the participants were represented using frequencies and percentages. Spearman rank correlation was used to analyze the relationship between PCOQ scores and participant characteristics. A P-value <0.05 was considered for all calculations. To examine the reliability of PCOQ, we calculated Cronbach’s alpha for each subscale and summary score. Cronbach’s alpha values ranging from 0.7 to 0.8 were considered acceptable. We evaluated the inter-item correlation to see how many domains assessed the topic. Hence, 0.2 to 0.4 was considered a good inter-item correlation [17]. Moreover, exploratory factors were analyzed using principal component analysis. Additionally, rotation ProMax with an eigenvalue of more than 1 was used to create eigenvalues of screen plots. However, variability of only equal or above 0.40 was included. To determine data eligibility for measures of factors, we calculated Kaiser-Meyer-Olkin of equal or more than 0.5 for sampling adequacy.

## Results

The content validity analysis showed a representativeness score of 97.3% and a clarity score of 95.2%, which indicated good agreement. As illustrated in Table 1, the majority of participants were males (61%). Additionally, 50.7% of the participants were 30 to 50 years old.

**Table 1:** Sociodemographic data of study participants.

Characteristic		N (%) (n = 300)
Gender	Male	183 (61.0)
	Women	117 (39.0)
	Total	300 (100.0)
Age (year)	<30	42 (14.0)
	30–50	152 (50.7)
	>50	106 (35.3)
	Total	300 (100.0)
Martial status	Married	258 (86.0)
	Single	32 (10.7)
	Widow	10 (3.3)
	Total	300 (100.0)
Education level	High school	196 (65.3)
	Bachelor	72 (24)
	Higher education	10 (3,3)
	Diploma	22 (7.3)
	Total	300 (100.0)

Table 2 illustrates the results of the internal consistency measures. The overall mean score of PCOQ was 3.3 with an SD of 1.27. The subscale scores ranged from 0.79 ± 1.2 to 9.2 ± 1.0. The individual items of the PCOQ score ranged from 0.59 ± 1.1 to 9.3 ± 1.3. The overall Arabic PCOQ questionnaire Cronbach’s alpha was 0.851, while for the subscales, it ranged between 0.68 and 0.93. Moreover, inter-item average correlation ranged from 0.7 to 0.95. Item-total correlation was more than 0.4 in the majority of the items. The feasibility of factor analysis (Bartlett test of sphericity) showed an acceptable value (Kaiser-Meyer-Olkin PCOQ = 0.5).

**Table 2:** Mean scores of participants on the scales and items along with correlations. **: High positive total item correlation.

Scale items	Mean score (SD)	95% CI	Item scale correlation	Item total correlation	Cronbach’s alpha
Overall PCOQ score	3.3 (1.27)	3.15–3.44			0.851
Current level	4.0 (2.61)	3.71–4.29			0.681
1	4.2 (3.6)	3.79–4.60	0.743	0.636	
2	3.24 (3.7)	2.82–3.65	0.793	0.660	
3	4.6 (3.6)	4.19–5.00	0.622	0.344	
4	4.0 (3.6)	3.59–4.40	0.705	0.525	
Desired level	0.94 (1.9)	0.72–1.15			0.912
5	0.94 (2.01)	0.71–1.16	0.909	0.696	
6	0.84 (1.8)	0.63–1.05	0.851	0.623	
7	1.0 (2.3)	0.74–1.26	0.941	0.828^**^	
8	1.0 (2.5)	0.71–1.28	0.880	0.819^**^	
Successful level	0.79 (1.2)	0.65–0.92			0.771
9	0.82 (1.2)	0.66–0.93	0.776	0.528	
10	0.59 (1.1)	0.46–0.71	0.629	0.323	
11	0.68 (1.4)	0.52–0.83	0.878	0.606	
12	1.0 (2.6)	0.70–1.29	0.898	0.806^**^	
Expected level	1.58 (2.6)	1.28–1.87			0.930
13	1.6 (2.7)	1.29–1.90	0.957	0.833^**^	
14	1.43 (2.8)	1.11–1.74	0.941	0.837^**^	
15	1.2 (2.4)	0.92–1.47	0.949	0.831^**^	
16	2.1 (3.3)	1.72–2.47	0.838	0.823^**^	
Important level	9.2 (1.0)	9.08–9.31			0.825
17	9.1 (1.2)	8.96–9.23	0.894	-0.431	
18	9.2 (1.3)	9.05–9.34	0.813	-0.716	
19	9.3 (1.2)	9.16–9.43	0.737	-0.716	
20	9.3 (1.3)	9.16–9.43	0.0798	-0.106	

**Figure 2:**
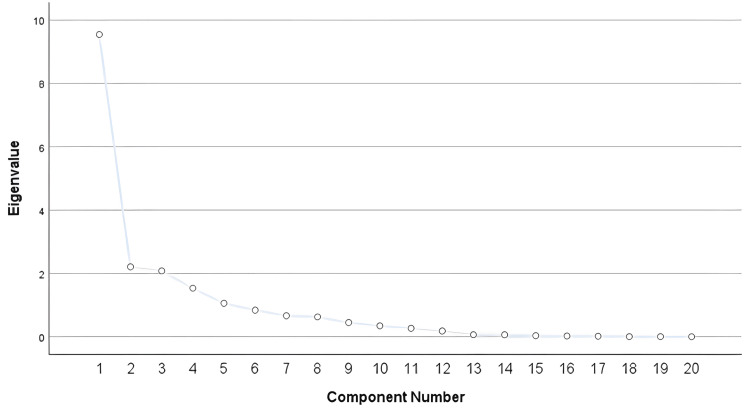
Scree plot of patient-centered outcomes questionnaire.

Exploratory factor analysis was performed with scree plots along with eigenvalue criteria to further explore the pattern in items and the most possible match adhering to the structure of the tool (Figure 2). Five components explained 82% of the variation in PCOQ with 15 components which identified 18 variations of the PCOQ tool.

Table 3 shows the result of factor loading of the PCOQ items. A factor loading of more than 0.2 was reported in PCOQ factors in 90% (18/20) of the factors.

**Table 3:** Factor loading of patient-centered outcomes questionnaire (PCOQ) for the one-factor structure.

Items	PCOQ factor loading
Current level	
Pain	0.576
Fatigue or tiredness	0.635
Emotional disorder	0.585
Impact on daily activities	0.547
Desired level	
Pain	0.666
Fatigue or tiredness	0.650
Emotional disorder	0.790
Impact on daily activities	0.850
Successful level	
Pain	0.595
Fatigue or tiredness	0.424
Emotional disorder	0.695
Impact on daily activities	0.843
Expected level	
Pain	0.886
Fatigue or tiredness	0.865
Emotional disorder	0.823
Impact on daily activities	0.799
Important level	
Pain	0.707
Fatigue or tiredness	0.260
Emotional disorder	0.274
Impact on daily activities	0.819

## Discussion

PCOQ is a self-report questionnaire that aims to assess four fundamental domains (pain, fatigue, distress, and interference) on an NRS ranging from 0 to 10 in the chronic pain population. It was successfully applied in this study on periodontitis patients [14]. The importance of this study arises from the fact that it is the first application of this questionnaire in Arabic and the first application in the field of dentistry. The PCOQ has been used in the medical field and successfully measured different types of chronic pain. For instance, fibromyalgia, a condition that causes musculoskeletal pain and approximately affects 2% of adults with chronic pain, was studied in different studies assessing the patient-centered treatment approach in such cases [18-20]. The result of this approach is promising; hence, its application in the dental field was inevitable. Moreover, the importance of the present article goes beyond this, as it is a translation of the PCOQ from English to Arabic, which will enable researchers to use the translated version to widen the scope of the questionnaire and make it available in an accurately translated Arabic version. Regarding the validation of the Arabic version of the PCOQ, content validity was implemented, and the analysis revealed an excellent representativeness and clarity score, indicating good agreement among the translation group.

The psychometric features of the Arabic questionnaire were tested among a sample of 300 people suffering from chronic periodontitis. We obtained satisfactory evidence on the psychometric qualities of the Arabic version of the PCOQ and internal consistency for the overall Arabic PCOQ (0.851). A study by O’Brien et al. in 2010 demonstrated a PCOQ value greater than 2.4 [14], while the present study reported a value less than 2. According to the patient-centered outcome model, this score indicates that the Saudi population with chronic periodontitis felt a low level of pain, tiredness, discomfort, and interference from daily life activity. This outcome implies that the translated questionnaire could be used in other Arab nations while retaining its reliable psychometric features. Furthermore, in this study, the current level of PCOQ was the least reliable domain compared with the different levels (desired level, successful level, and important level). In the future, more research is needed to assess the reproducibility of the tool in terms of test-retest reliability of the instrument.

## Conclusions

The PCOQ underwent a well-structured methodology to satisfy the best standards for translation. After the utilization of these standards, the Arabic version of the PCOQ satisfied the requirements, and it is now ready to be used. Hence, this validated Arabic version of the PCOQ can serve as an important patient-centered outcome measure of care in Arab nations. Nonetheless, this instrument can provide insight for healthcare professionals, policymakers, and managers to improve patients’ satisfaction and improve healthcare system delivery.
